# EEF1D overexpression promotes osteosarcoma cell proliferation by facilitating Akt-mTOR and Akt-bad signaling

**DOI:** 10.1186/s13046-018-0715-5

**Published:** 2018-03-06

**Authors:** Dong-dong Cheng, Shi-jie Li, Bin Zhu, Shu-min Zhou, Qing-cheng Yang

**Affiliations:** 1 Department of Orthopedics, Shanghai Jiao Tong University Affiliated Sixth People’s Hospital, No. 600, Yishan Road, Shanghai, 200233 China; 2 Institution of microsurgery for limbs, Shanghai Jiao Tong University Affiliated Sixth People’s Hospital, No. 600, Yishan Road, Shanghai, 200233 China

**Keywords:** EEF1D, Proliferation, Akt-mTOR signaling pathway, Akt-bad signaling pathway, Osteosarcoma

## Abstract

**Background:**

Dysregulation of eukaryotic translation elongation factor 1 delta (EEF1D) in cancers has been reported; however, the role and mechanisms of EEF1D in osteosarcoma remain poorly understood. The aim of this study is to investigate the expression and role of EEF1D in osteosarcoma and to elucidate its underlying mechanisms.

**Methods:**

The expression of EEF1D in osteosarcomas and cell lines was evaluated by qRT-PCR, Western blotting and immunohistochemistry. EEF1D knockdown using small interfering RNA (siRNA) was employed to analyze the role of EEF1D in osteosarcoma cell proliferation and cell cycle progression. The host signaling pathways affected by EEF1D knockdown were detected using PathScan® intracellular signaling array kit.

**Results:**

The expression of EEF1D was found to be up-regulated in human osteosarcoma tissues and cell lines. Its expression was positively correlated with Enneking stage and the tumor recurrence. EEF1D knockdown inhibited osteosarcoma cell proliferation, colony-forming ability, and cell cycle G2/M transition in vitro. In addition, EEF1D knockdown decreased the levels of phospho-Akt, phospho-mTOR, and phospho-Bad proteins.

**Conclusions:**

EEF1D is upregulated in osteosarcoma and plays a tumor promoting role by facilitating Akt-mTOR and Akt-Bad signaling pathways. Accordingly, EEF1D is a potential target for cancer therapy.

**Electronic supplementary material:**

The online version of this article (10.1186/s13046-018-0715-5) contains supplementary material, which is available to authorized users.

## Background

Osteosarcoma, the most common primary skeletal tumor in children and adolescents, is characterized by the direct formation of immature bone or osteoid tissue by tumor cells [1]. Osteosarcoma frequently affects the metaphysis of long bones, particularly the distal femur, proximal tibia, and proximal humerus [2]. With the introduction of neoadjuvant chemotherapy, the 5-year overall survival rate of osteosarcoma has climbed from 20% to 75% [3, 4]. However, despite research and advances in chemotherapy regimens, the prognosis of patients with osteosarcoma remains highly variable and is often dismal during the last three decades owing to chemotherapy insensitivity [5]. Therefore, elucidating the underlying molecular mechanisms of osteosarcoma is critical for developing new therapeutic strategies to improve the prognosis of patients.

The process of eukaryotic protein synthesis traditionally proceeds in three phases: initiation, elongation, and termination [6]. The function of the eukaryotic elongation factor 1 (eEF1) complex has been well investigated in translation elongation [7–9]. Apart from their function in translation elongation, components of the eEF1 complex have been implicated in tumorigenesis. Deregulation of eukaryotic translation elongation factor 1 alpha (eEF1A) has been implicated in breast carcinoma [10], and this has been attributed to eEF1A-mediated activation of sphingosine kinase 1 (SK1) [11]. Veremieva et al. examined the expression of eEF1 complex subunits in cardioesophageal carcinoma and correspondingly paired normal tissues, and found that at least one eEF1 component was overexpressed in 72% of tumor tissues [12]. Similarly, overexpression of eEF1B2 was detected in human lung cancer, signifying its important role in tumorigenesis [13].

Eukaryotic translation elongation factor 1 delta (EEF1D), another subunit of the eEF1 complex, mediates the elongation process after the translation initiation complex has formed [14]. The translation elongation rate in mammalian cells is regulated by activation of the cyclin-dependent kinase 1 (CDK1) during the mitotic (M) phase of the cell cycle [15]; and EEF1D was reported to be phosphorylated by CDK1 at Ser-133 [16]. Recently, the role of EEF1D in tumors, including chondrosarcoma [17] and papillary renal cell carcinoma, [18] has been investigated, and mutation of EEF1D may contribute to the tumorigenesis of Papillary renal cell carcinoma. However, the role and underlying molecular mechanisms of EEF1D in osteosarcoma remain unclear.

In this study, we investigated the expression of EEF1D in human osteosarcoma samples and osteoblast cell lines. EEF1D was found to be overexpressed in osteosarcoma cell lines and tissues. The functions and underlying mechanisms of EEF1D were further investigated in osteosarcoma cells. Our results demonstrated that knockdown of EEF1D hindered osteosarcoma cell proliferation and colony-forming ability by inhibiting the G2/M cell cycle transition. In order to understand the potential mechanism of EEF1D, we detected the host signaling pathways affected by EEF1D using PathScan® intracellular signaling array kit. We found that reduction in EEF1D downregulated the Akt-mTOR and Akt-Bad signaling pathways in osteosarcoma cells. Notably, we found that EEF1D expression correlated positively with the Enneking stages and recurrence of osteosarcoma. To the best of our knowledge, this is the first report that EEF1D overexpression played an oncogenic role in osteosarcoma.

## Methods

### Cell lines and culture conditions

Three osteosarcoma cell lines (MNNG/HOS, U2OS and MG63) and human osteoblast cell line (hFOB 1.19) were used in this study. All cell lines were obtained from the Cell Bank of the Chinese Academy of Sciences (Shanghai, China). The MNNG/HOS and MG63 cells were cultured in Dulbecco’s modified Eagle’s medium (DMEM), emented with 10% fetal bovine serum (Biowest, South America), 100 U/mL penicillin (Sigma-Aldrich, St Louis, MO, USA), and 100 mg/mL streptomycin (Sigma-Aldrich). U2OS cells were cultured in Roswell Park Memorial Institute (RPMI)-1640 medium, supplemented with 10% fetal bovine serum, 100 U/mL penicillin, and 100 mg/mL streptomycin. hFOB 1.19 cells were cultured in a 1:1 mixture of Ham’s F12 medium and Dulbecco’s modified Eagle’s medium with 2.5 mM L-glutamine supplemented with 100 U/mL penicillin, 100 U/mL streptomycin, 0.3 mg/ml G418 (Sigma) and 10% FBS.

### Human osteosarcoma samples

In the period from 2014 to 2015, 50 osteosarcoma patients were treated at the Shanghai Jiao Tong University Affiliated Sixth People’s Hospital. There are 31 males and 19 females. The median age of the patients was 18 years old (range: 8–64 years). 24 patients had local recurrence after en bloc resection of the primary tumor. The follow-up period ranged from 21 to 36 months, and the median time was 28.2 months. 28 patients developed pulmonary metastasis after the surgery. No other metastatic site was found. They received primary surgical treatment, and preoperative and postoperative neoadjuvant therapy. For each patient, an osteosarcoma sample and a corresponding adjacent non-tumor tissue sample were obtained during surgery. The samples were immediately frozen in liquid nitrogen after resection and stored at − 80 °C. Ethics approval was obtained from the local hospital ethics committees and written informed consent was obtained from each patient prior to sample collection (YS-2016-064, 24 February 2016).

### RNA extraction and qRT-PCR analysis

Total RNA from human tissue samples and cultured cells was purified using TRIzol reagent (Invitrogen, Carlsbad, CA, USA). cDNA was synthesized using PrimeScript RT Reagent Kit (Takara, Shiga, Japan). qRT-PCR was performed using SYBR Green Premix Ex Taq (Takara, Shiga, Japan) on an ABI 7500 PCR system (Applied Biosystems). All reactions were performed in triplicate in a final reaction volume of 10 μL. The primer sequences used were: EEF1D forward: 5′-ACAGACCCAGCACGTATCTC-3′, EEF1D reverse: 5′-CCAGCAGGATGGAGGACTTG-3′, β-actin forward: 5′-TTGTTACAGGAAGTCCCTTGCC-3′, and β-actin reverse: 5′-ATGCTATCACCTCCCCTGTGTG-3′. Relative quantification was determined using the Δ Ct method in the qRT-PCR.

### Protein extraction and western blotting analysis

Lysates were prepared from cultured cells using T-PER Protein Extraction Reagent (Thermo Fisher Scientific) containing PhosSTOP (Roche, Basel, Switzerland) and Complete Mini protease inhibitor cocktail (Roche, Basel, Switzerland). Equal amounts of proteins were electrophoresed and transferred onto polyvinylidene difluoride (PVDF) membranes (Millipore, Billerica, MA, USA). After blocking in 5% nonfat milk, the membranes were incubated with the following primary antibodies: EEF1D (1:500, Proteintech) [19], mTOR (total, 1:1000; Cell Signaling Technology), phospho-mTOR (Ser2448, 1:1000; Cell Signaling Technology), Akt (total, 1:1000; Cell Signaling Technology), phospho-Akt (Thr308, 1:1000; Cell Signaling Technology), Bad (total, 1:500; BBI Life Sciences), phospho-Bad (Ser112, 1:500; BBI Life Sciences), or β-actin (1:20,000, Sigma-Aldrich). Anti-rabbit IgG (1:5000, Sigma-Aldrich) was used as the secondary antibody. Visualization was performed with SuperSignal West Femto Maximum Sensitivity Substrate (Thermo Fisher Scientific).

### Small interfering RNA (siRNA) and plasmid transfection

Human EEF1D siRNA (si-EEF1D) and a nonspecific control siRNA (si-NC) were synthesized by RiboBio (Guangzhou, China). pcDNA 3.1 and pcDNA 3.1-EEF1D were synthesized by Bioworld (Nanjing, China). The siRNAs and plasmids were transfected into cells using Lipofectamine 2000 reagent (Invitrogen) following the manufacturer’s protocol. The siRNA sequence targeting EEF1D was—5′-TGACGAGGATGATGACATT-3′. The nonspecific control for siRNA was purchased from Ribobio (Guangzhou, China). The siRNA sequence targeting control siRNA was—5′-GATCATACGTGCGATCAGA-3′. Cell proliferation assay, cell cycle analysis, RNA extraction, and western blotting were performed 48 h after transfection.

### Cell proliferation and colony-formation assays

At 48 h after transfection, cells were trypsinized and re-seeded into 96-well plates (3000 cells/well) for the cell proliferation assay. Then, 10 μL of Cell Counting Kit-8 (CCK-8) (Dojindo, Kumamoto, Japan) solution was added to each well and absorbance at 450 nm was measured after 2 h of incubation. All experiments were performed in triplicate for each condition and repeated twice. For the colony formation assay, transfected MNNG/HOS, U2OS, or MG63 cells (1 × 10^3^ cells/well) were cultured in 6-well plates for 10 d and subjected to 100% methanol fixation and 0.1% crystal violet staining for 30 min each. The cell colonies were counted and all assays were performed independently in triplicate.

### Cell cycle analysis

Forty-eight hours after transfection, cells were collected and fixed with 70% ethanol. Cells were then stained with 50 μg/mL propidium iodide (PI; Kaiji, China) containing RNaseI (Kaiji, China) and analyzed using a FACSCalibur flow cytometer (BD Biosciences, San Jose, CA). The results were evaluated using ModFit (BD Biosciences). Assays were performed three times independently.

### Intracellular signaling arrays

Forty-eight hours after transfection, MNNG/HOS and U2OS cells were harvested and lysed for 5 min on ice using 0.1 mL of cell lysis buffer included in the PathScan Antibody Array Kit, which contains a cocktail of protease inhibitors. The lysates were centrifuged at 10,000 *g* at 4 °C for 10 min. Intracellular signaling molecules were detected using a PathScan® intracellular signaling array kit (Cell Signaling Technology, #7744) according to manufacturer’s instructions. Fluorescent images of the slides were captured using Odyssey® Infrared Imaging System (LI-COR) and spot intensities were quantified using Image Studio™ analysis software.

### Immunohistochemistry (IHC)

Standard procedures for IHC staining of osteosarcoma samples were described previously [20]. Briefly, 4 μm sections were cut form paraffin-embedded osteosarcoma samples. The sections were de-paraffinized in xylene, and then heated at 60 °C for 20 min in EDTA buffer (pH 9.0) for antigen retrieval. Endogenous peroxidase activity was blocked by incubation in 0.3% hydrogen peroxide for 10 min. Immunostaining was performed by incubation at 37 °C with anti-EEF1D primary antibody (1:100; Proteintech, China). Sections were rinsed three times in phosphate-buffered saline (PBS) and incubated with EnVision staining kit (DAKO, Denmark) for 30 min. After three additional washes in PBS, color development was achieved over 3–10 min in a humidified chamber at room temperature using 3,3′-diaminobenzidine (DAB). Sections were counterstained with hematoxylin and dehydrated in a graded ethanol series (70%, 90%, and 100%). For negative control sections, the primary antibody was substituted with PBS. IHC signal intensities were scored as follows: 0 (no staining), 1 (staining in < 1% cells), 2 (staining in 1%–10% cells), or 3 (staining in > 10% cells). The samples classified as 0 and 1 were considered negative, while those classified as 2 and 3 were considered positive. Assessment of IHC staining was independently performed by two expert pathologists. A third pathologist was consulted in case a discrepancy raised. Any discordance was resolved through discussion until consensus was reached.

### Statistical evaluation

Data were compiled and analyzed using SPSS version 21.0 (SPSS Inc., Chicago, IL, USA). The differences between the groups were compared using two-tailed Student’s *t*-test. The correlations between IHC and clinicopathologic parameters were determined using chi-square test. *P* < 0.05 was considered significant.

## Results

### EEF1D is overexpressed in osteosarcoma cell lines and human osteosarcoma tissue samples

**Fig. 1:**
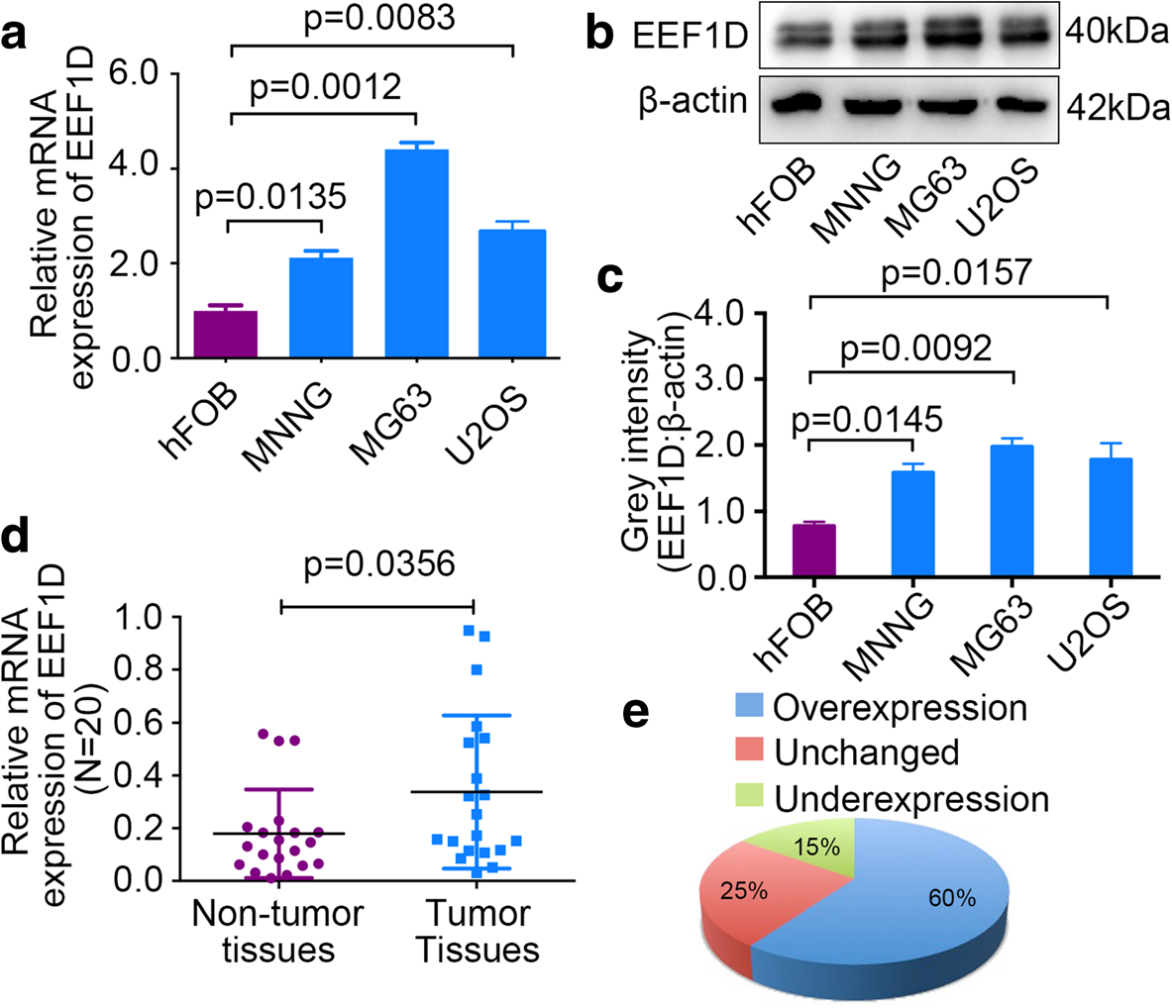
EEF1D is upregulated in osteosarcoma tissues and cell lines. **a**, **b**, **c** EEF1D mRNA and protein expression levels in osteosarcoma cell lines (MNNG/HOS, MG63 and U2OS) and human normal osteoblast cell line (hFOB 1.19) were measured by qRT-PCR and western blotting, respectively. **d**, **e** EEF1D expression levels were measured in 20 pairs of osteosarcoma and corresponding adjacent non-tumor tissues. For qRT-PCR, EEF1D expression was normalized to β-actin

To determine the role of EEF1D in osteosarcoma, we first examined EEF1D mRNA and protein expression levels in osteosarcoma and osteoblast cell lines by qRT-PCR and western blotting analysis. Compared with the osteoblast cell line, the osteosarcoma cell lines expressed significantly higher levels of both EEF1D mRNA and protein (Fig. 1a-c). Further, EEF1D mRNA expression levels were investigated in 20 paired osteosarcoma and adjacent non-tumor tissue samples. As shown in Fig. 1d-e, EEF1D expression was significantly upregulated in 60% (12/20) of osteosarcoma tissues compared with that in adjacent non-tumor tissues. These results show that EEF1D is overexpressed in osteosarcoma and may play an important role in osteosarcoma tumorigenesis, thus, warranting further investigation.

### Knockdown of EEF1D inhibits osteosarcoma cell proliferation in vitro

**Fig. 2:**
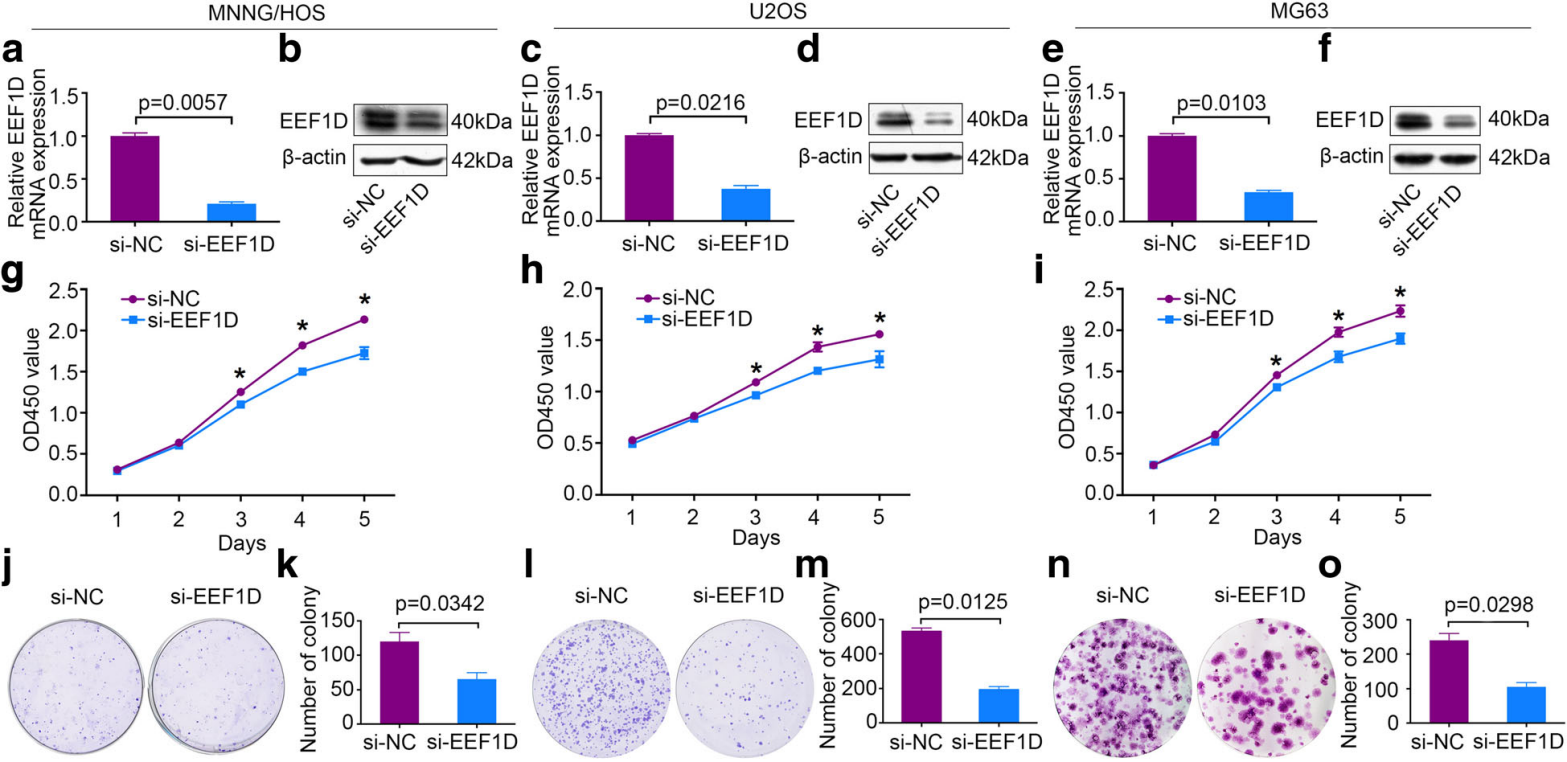
EEF1D Knockdown inhibits osteosarcoma cell growth in vitro. **a**-**f** Expression levels of EEF1D mRNA and protein in MNNG/HOS, U2OS and MG63 cells were measured after EEF1D siRNA (si-EEF1D) transfection by qRT-PCR and western blotting, respectively. **g**-**i** Cell Counting Kit-8 (CCK-8) assays were performed to measure cell proliferation after siRNA transfection. **j**-**o** Colony-formation assays were performed for EEF1D-silenced osteosarcoma and control cells. Data are representative of results from three independent experiments. * *P* < 0.05. For qRT-PCR, EEF1D expression was normalized to β-actin

To explore the functional significance of EEF1D in osteosarcoma, we used si-EEF1D to knockdown EEF1D in osteosarcoma cells. In MNNG/HOS, U2OS and MG63 cells, EEF1D mRNA and protein expression levels were significantly reduced after transfection with si-EEF1D (Fig. 2a-f), validating the knockdown effects. We examined the EEF1D expression on day 5 in MNNG,MG63 and U2OS cells. The results indicate that EEF1D expression remained inhibited 5 days after transfection with si-EEF1D (Additional file 1: Figure S1). A five-day growth curve analysis using CCK-8 assay showed that knockdown of EEF1D significantly inhibited the growth of osteosarcoma cells (Fig. 2gi–i). Colony-forming assays were performed to determine the colony-forming capacity of osteosarcoma cells after EEF1D knockdown. We found that the number and size of colonies were both significantly decreased in the EEF1D-knockdown group compared with those in the control group (Fig. 2j-o). In addition, the cell apoptosis assay showed that knockdown of EEF1D did not affect the cell apoptosis in osteosarcoma cells (Additional file 2: Figure S2). These results demonstrate that EEF1D plays a tumor promoting role in osteosarcoma.

### Knockdown of EEF1D inhibits osteosarcoma cell cycle G2/M transition

**Fig. 3:**
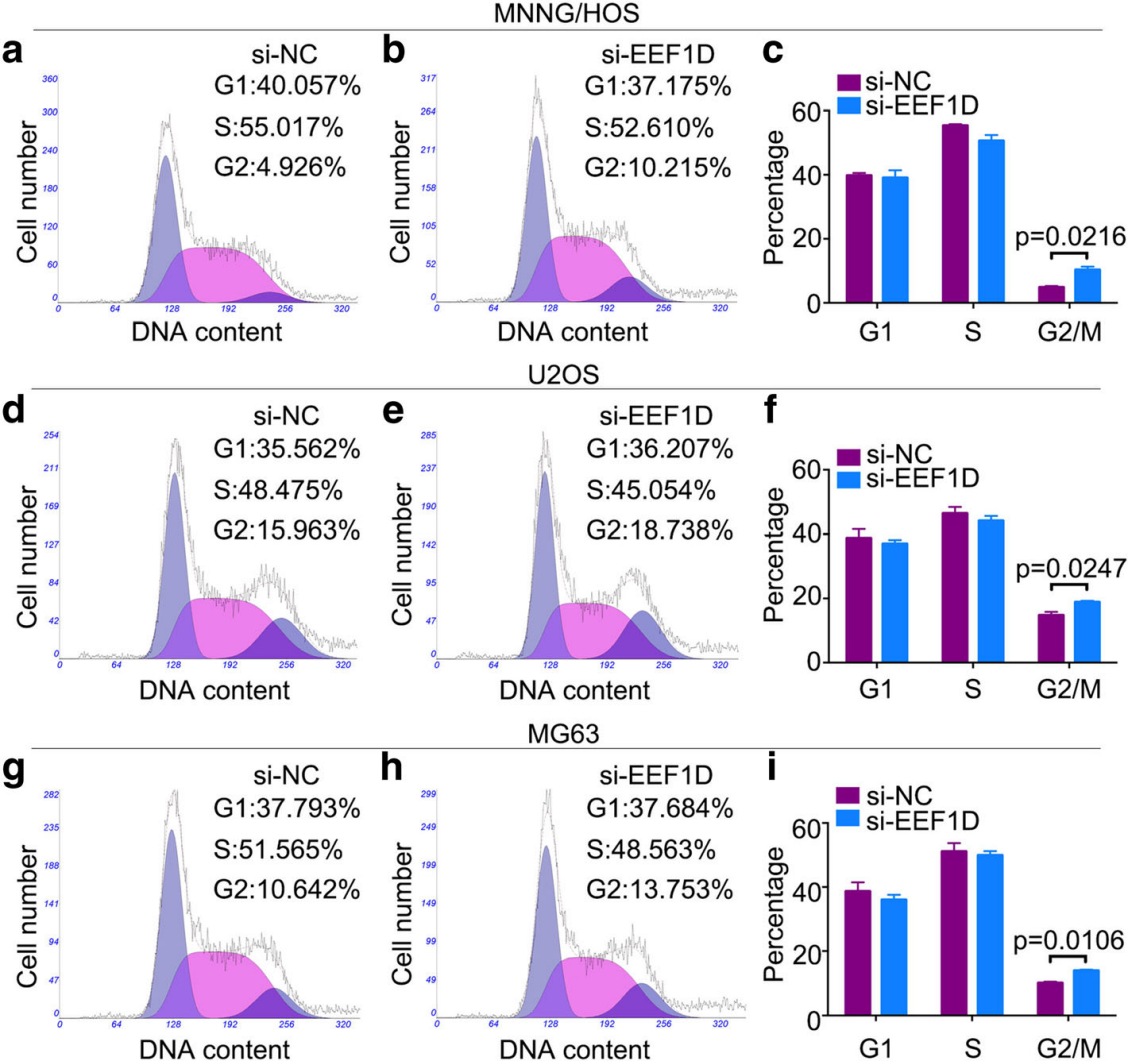
EEF1D Knockdown inhibits osteosarcoma cell cycle G2/M transition. Representative images of the cell cycle assays in MNNG/HOS (**a**, **b**), U2OS (**d**, **e**) and MG63 cells (**g**, **h**) after transfection with nonspecific control siRNA (si-NC) or EEF1D siRNA (si-EEF1D). **c**, **f**, **i** Diagrams showing the results of cell cycle assay in MNNG/HOS, U2OS and MG63 cells

Changes in the cell cycle profile following EEF1D knockdown were analyzed by flow cytometry. The percentage of cells in the G2/M of the cell cycle was significantly increased for si-EEF1D-transfected cells compared with that of si-NC-transfected cells (Fig. 3a-i). However, no significant difference was found in the percentage of cells in the G1 or S phases after EEF1D knockdown. Together, the data shows that EEF1D knockdown attenuates osteosarcoma cell proliferation by inhibiting the G2/M transition.

### EEF1D knockdown modulates the Akt-mTOR and Akt-bad signaling pathways

**Fig. 4:**
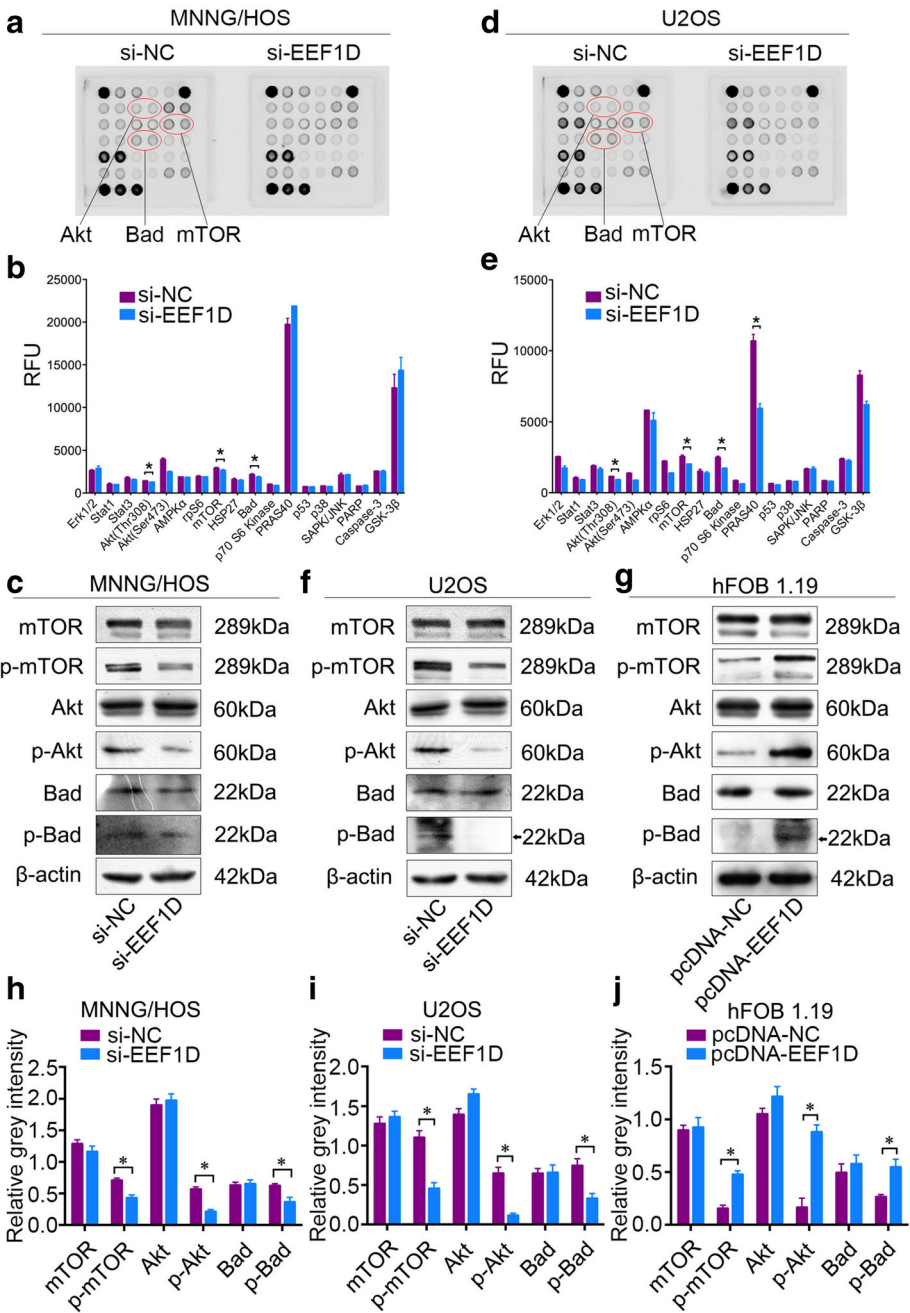
EEF1D Knockdown inhibits Akt-mTOR and Akt-Bad signaling pathways in osteosarcoma cells. **a**, **b** MNNG/HOS and U2OS cell extracts were prepared and analyzed using PathScan® intracellular signaling array kit. Images were captured with Odyssey® Infrared Imaging System (LI-COR). **c**, **d** Quantification of results from MNNG/HOS and U2OS cells shown in (**a**, **b**) **e**, **f** Western blotting analysis of Akt-mTOR and Akt-Bad signaling pathway molecules in MNNG/HOS and U2OS cells transfected with nonspecific control siRNA (si-NC) or EEF1D siRNA (si-EEF1D). **g** Western blotting analysis of Akt-mTOR and Akt-Bad signaling pathway molecules, including mTOR, Akt, and Bad, in hFOB 1.19 cells transfected with pcDNA 3.1 or pcDNA 3.1-EEF1D. Quantification of results in (**e**, **f**, **g**) were shown in (**h**, **i** and **j**) respectively

To determine the potential mechanism by which EEF1D affects the proliferation of osteosarcoma cells, we screened for changes of intracellular signaling molecules resulted from EEF1D knockdown in MNNG/HOS and U2OS cells. A slight decrease in the phosphorylation of Akt-Thr308, mTOR-Ser2448 and Bad-Ser112 was detected in these osteosarcoma cell lines (Fig. 4a-d). We further examined whether EEF1D knockdown could affect the Akt-mTOR and Akt-Bad signaling pathways in osteosarcoma cells using Western blotting analysis, and the results revealed that EEF1D knockdown inhibited the phosphorylation of Akt, mTOR, and Bad (Fig. 4e-i). To further substantiate those findings, we overexpressed EEF1D in hFOB 1.19 cells and examined the change of Akt-mTOR and Akt-Bad signaling pathways. The western blotting assay confirmed the overexpression (Additional file 3: Figure S3). The results showed that overexpression of EEF1D increased the phosphorylation of Akt, mTOR and Bad (Fig. 4g, j). Taken together, out data support that EEF1D may play an important role in osteosarcoma cell growth by enhancing the Akt-mTOR and Akt-Bad signaling pathways.

### EEF1D expression correlates with osteosarcoma Enneking stage and tumor recurrence

**Fig. 5:**
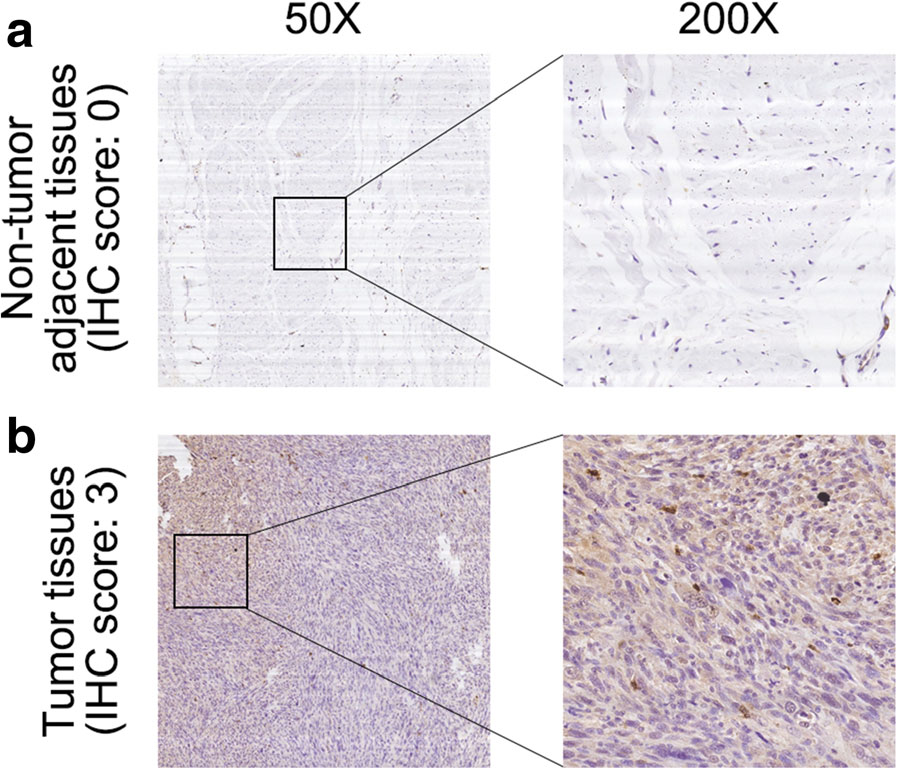
Clinical significance of EEF1D in osteosarcoma patients. **a** Representative IHC image of EEF1D in non-tumor tissues. **b** Representative IHC image of EEF1D in osteosarcoma. Original magnification: 50×, 200×

To further determine the clinicopathological significance of EEF1D in osteosarcoma, we performed IHC analysis of EEF1D in 50 human osteosarcoma tissue samples and the corresponding non-tumor tissues. Representative IHC images showing the expression of EEF1D in osteosarcoma and adjacent non-tumor tissues are shown in Fig. 5. From the Figure, we can see in osteosarcoma tissues the nucleus and cytoplasm are positively stained. However, the adjacent nontumor tissues have a negative staining of EEF1D. Correlations between EEF1D expression levels evaluated by IHC and clinicopathological characteristics of osteosarcoma patients are summarized in Table 1. EEF1D expression levels were higher in osteosarcoma tissue samples than in the corresponding non-tumor tissues (*P* = 0.018). The expression levels of EEF1D were higher in patients at a clinically advanced Enneking stage than in patients at an early stage (*P* = 0.007). Further analysis revealed that EEF1D levels were positively correlated with recurrence (*P* = 0.013). No correlation was found between EEF1D and any of the other factors, including gender, age, tumor location, tumor necrosis rate, cortical destruction and metastasis. Taken together, these results indicate that EEF1D is upregulated in osteosarcoma and potentially plays an important role in osteosarcoma progression.

**Table 1:** Correlation analyses of EEF1D protein expression in relation to clinicopathologic variables of 50 patients with osteosarcoma

Clinicopathologic parameters	Number of cases	EEF1D expression level		*p* value
		Negative	Positive	
Gender				0.513
Male	31	11	20	
Female	19	6	13	
Age (years)				0.279
< 18	28	11	17	
≥ 18	22	6	16	
Location				0.620
Femur	27	8	19	
Tibia	10	3	7	
Elsewhere	13	6	7	
Tumor necrosis rate (%)				0.326
< 90	44	14	30	
≥ 90	6	3	3	
Cortical destruction				0.554
Yes	43	15	28	
No	7	2	5	
Recurrence				0.013^*^
Yes	24	4	20	
No	26	13	13	
Metastasis				0.112
Yes	28	7	21	
No	22	10	12	
Ennecking stage				0.005^*^
II	39	17	22	
III	11	0	11	

^*^P < 0.05

## Discussion

In this study, we demonstrated that EEF1D, a subunit of the eEF1 complex, was upregulated in osteosarcoma cell lines and clinical tumor samples in comparison with the corresponding adjacent non-tumor tissues. Knockdown of EEF1D impaired osteosarcoma cell proliferation and colony-forming ability, and led to G2/M cell cycle arrest. These results indicate that EEF1D plays a critical role in osteosarcoma cell growth and acts as an oncogene in osteosarcoma. A proteomic analysis of adriamycin-resistant variants of the DLKP lung cancer cell line revealed that EEF1D levels correlated with the invasive potential of these cells [21]. Joseph et al. reported that *EEF1D* was a novel cadmium-responsive proto-oncogene [22]. De Bortoli et al. found that overexpression of EEF1D was adversely associated with the outcome of medulloblastoma [23]. Another comparative proteomics analysis of differentially-expressed proteins between Chinese left- and right-sided colon cancer showed that EEF1D expression was higher in the right-sided colon cancer [24]. In the present study, we found that the expression of EEF1D, as indicated by IHC staining, was positively correlated with osteosarcoma recurrence and Enneking stage. Our findings are consistent with those previous reports.

Interestingly, it was previously reported that EEF1D down-regulation promoted an increase in the number of cells at G0/G1-phase in an oral square cell carcinoma model, and that EEF1D knockdown significantly decreased cell proliferation, which were concomitant with a decrease in cyclin D1 expression and RB phosphorylation [25]. Considering the well-established role of cyclin D1/cdk4 in G1/S transition, those observations were not surprising. On contrary, the G2/M transition is regulated by cyclin B1/Cdc2 activity; mitosis follows DNA replication in the G2 phase of the cell-cycle after the mitotic Cdk1(cdc2) is activated. The G2 checkpoint allows the cell to repair DNA damage before entering mitosis [26]. Accordingly, DNA damage that occurs in a cell with a defective G1 checkpoint or dysregulated DNA replication commonly results in G2-M arrest. In addition, it was demonstrated that cyclin D1 depletion could trigger the G2/M arrest of HeLa (human cervical cancer cell) and HEK293 (human embryo kidney cell) [27]. We found that in OS cells, EEF1D knockdown resulted in G2/M arrest, suggesting EEF1D may affect cell cycle progression in osteosarcoma via different mechanisms. Whether EEF1D directly or indirectly affects CyclinD1/Cdk4 or Cyclin B1/Cdk1 activity in osteosarcomas remains to be investigated.

Mechanistically, it has been reported that EEF1D is a direct substrate for casein kinase 2 (CK2), an important regulator of cell cycle progression, apoptosis, and transcription [28, 29]. Wu et al. [30] reported the interaction of EEF1D with siah E3 ubiquitin protein ligase 1 (SIAH-1); and overexpression of EEF1D resulted in an increase in SIAH-1 levels through the inhibition of auto-ubiquitination and degradation of SIAH-1 in Hela cells. In the present study, we investigated the potential mechanisms underlying the role of EEF1D in osteosarcoma. We used the PathScan® intracellular signaling array kit to identify host signaling pathways that were affected by EEF1D knockdown. We observed that EEF1D knockdown led to a slight decrease in the phosphorylation of some components of the Akt-mTOR and Akt-Bad pathways, including Akt, mTOR, and Bad. Western blotting analysis further confirmed that knockdown of EEF1D inhibited the phosphorylation of Akt, mTOR and Bad. Considering that overexpression of EEF1D resulted in an increase in SIAH-1 level [30], and that Siah-1-interacting protein (CacyBP/SIP), a component of a novel ubiquitinylation pathway affects the proliferation of human glioma cells by regulating phospho-Akt (p-Akt) [31], we propose that EFF1D might regulate Akt-mTOR and Akt-Bad signaling pathways through interacting with SIAH-1.

Akt-mTOR and Akt-Bad are important intracellular signaling pathways, and are known to be closely associated with the progression of tumors, including osteosarcoma. A recent study showed that activation of the phosphoinositide 3-kinase/protein kinase B (PKB), also known as Akt/mammalian target of rapamycin (PI3K/Akt/mTOR) signaling pathway correlated with tumor progression and reduced patient survival [32]. Yu et al. reported that microRNA 21 (miR-21) silencing enhanced autophagic cell death by targeting the phosphatase and tensin homolog (PTEN) through inhibition of the PI3K/Akt/mTOR pathway [33]. In addition, it was reported that the phosphorylation status of the B-cell lymphoma 2 (Bcl-2)-associated death promoter (BAD) protein and BAD-mediated apoptotic pathway influenced the chemosensitivity of cancer cells and was associated with the development of cancers, including ovarian, breast, and colon cancer [34–36]. Song et al. reported that p53 suppressed osteosarcoma cell growth, metastasis, and angiogenesis through inhibition of the PI3K/Akt/mTOR signaling pathway [37]. Upon phosphorylation, activated mTOR contributed to osteosarcoma cellular transformation and poor prognosis [38]. Peng et al. reported that curcumin-loaded nanoparticles enhanced apoptotic cell death of osteosarcoma cells through inhibition of the Akt-Bad signaling pathway [39]. Our results suggest that EEF1D exerts its effect on osteosarcoma by promoting Akt-mTOR and Akt-Bad signaling pathways, which could be the mechanism by which EEF1D promotes tumor progression.

## Conclusions

In conclusion, we demonstrate for the first time that EEF1D is upregulated in human osteosarcoma cell lines and clinical tumor samples. High expression of EEF1D is positively correlated with Enneking stage and the recurrence of osteosarcoma, and facilitates osteosarcoma cell proliferation. Mechanistically, EEF1D exerts oncogenic effects by maintaining the Akt-mTOR and Akt-Bad signaling pathways in osteosarcoma. Overall, our data provide evidence that EEF1D is a potential therapeutic target for osteosarcoma.

## Additional files

Additional file 1: **Figure S1.** The effect of si-EEF1D on osteosarcoma cells 5 days after transfection. Western blotting was used to detect the expression of EEF1D after transfection with si-EEF1D in MNNG/HOS, MG63 and U2OS cell lines. (TIFF 1346 kb)

Additional file 2: **Figure S2.** The effect of EEF1D Knockdown on osteosarcoma cell apoptosis in vitro. Cell apoptosis assays were used to detect the cell apoptosis after transfection with si-EEF1D in MNNG/HOS and U2OS cell lines. Statistical analysis was performed using Student’s *t* test (*n* = 3). *P < 0.05. (TIFF 1169 kb)

Additional file 3: **Figure S3.** The effect of EEF1D overexpression on hFOB 1.19 cells after transfection with pcDNA-EEF1D. Western blotting was used to detect the expression of EEF1D after transfection with pcDNA-EEF1D and pcDNA-NC in hFOB 1.19 cells. (TIFF 1031 kb)

## Abbreviations

ATCC: American Type Culture Collection
BAD: B-cell lymphoma 2 (Bcl-2)-associated death promoter
CCK-8: Cell Counting Kit-8
CDK1: Cyclin-dependent kinase 1
CK2: Casein kinase 2
DMEM: Dulbecco’s modified Eagle’s medium
eEF1: eukaryotic elongation factor 1
eEF1A: eukaryotic translation elongation factor 1 alpha
EEF1D: eukaryotic translation elongation factor 1 delta
IHC: immunohistochemistry
OSCC: Oral squamous cell carcinoma
PCDF: Polyvinylidene difluoride
PKB: Phosphoinositide 3-kinase/protein kinase B
PTEN: Phosphatase and tensin homolog
RPMI: Roswell Park Memorial Institute
SIAH-1: Siah E3 ubiquitin protein ligase 1
siRNA: small interfering RNA
SK1: Sphingosine kinase 1
