# Supplementary figures and images for: EEF1D overexpression promotes osteosarcoma cell proliferation by facilitating Akt-mTOR and Akt-bad signaling
Source: J Exp Clin Cancer Res. 2018 Mar 6;37:50. doi: 10.1186/s13046-018-0715-5 (PMC5839064; doi:10.1186/s13046-018-0715-5)

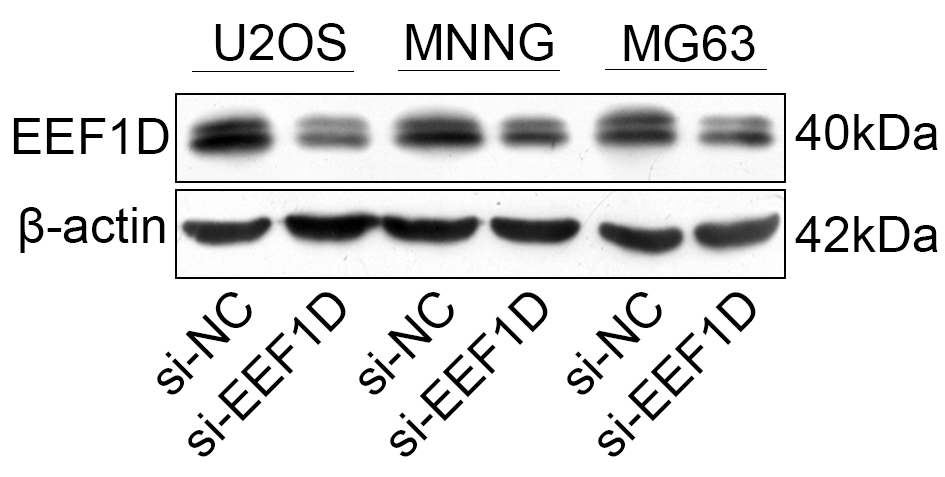

Supplement: Supplementary file 1 — Figure S1. The effect of si-EEF1D on osteosarcoma cells 5 days after transfection. Western blotting was used to detect the expression of EEF1D after transfection with si-EEF1D in MNNG/HOS, MG63 and U2OS cell lines. (TIFF 1346 kb) [file 13046_2018_715_MOESM1_ESM.tif]

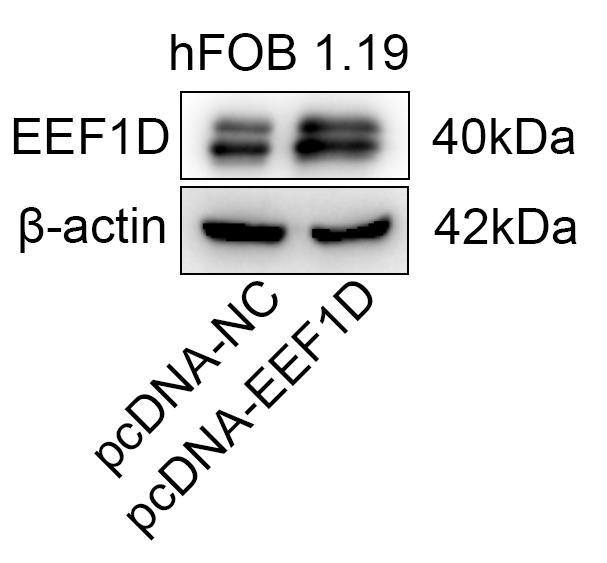

Supplement: Supplementary file 3 — Figure S3. The effect of EEF1D overexpression on hFOB 1.19 cells after transfection with pcDNA-EEF1D. Western blotting was used to detect the expression of EEF1D after transfection with pcDNA-EEF1D and pcDNA-NC in hFOB 1.19 cells. (TIFF 1031 kb) [file 13046_2018_715_MOESM3_ESM.tif]
